# The Role of Neutrophils in the Pathogenesis of Chronic Lymphocytic Leukemia

**DOI:** 10.3390/ijms23010365

**Published:** 2021-12-29

**Authors:** Malgorzata Wachowska, Alicja Wojciechowska, Angelika Muchowicz

**Affiliations:** 1 Department of Laboratory Diagnostics and Clinical Immunology of Developmental Age, Medical University of Warsaw, 02-097 Warsaw, Poland; malgorzata.wachowska@wum.edu.pl; 2 Department of Clinical Immunology, Medical University of Warsaw, 02-097 Warsaw, Poland; alicja.wojciechowska@wum.edu.pl; 3 Laboratory for Cellular and Genetic Therapies, Medical University of Warsaw, 02-097 Warsaw, Poland; 4 Department of Immunology, Medical University of Warsaw, 02-097 Warsaw, Poland

**Keywords:** neutrophils, CLL, NETs

## Abstract

Tumor-associated neutrophils appear to be a crucial element of the tumor microenvironment that actively participates in the development and progression of cancerous diseases. The increased lifespan, plasticity in changing of phenotype, and functions of neutrophils influence the course of the disease and may significantly affect survival. In patients with chronic lymphocytic leukemia (CLL), disturbances in neutrophils functions impede the effective immune defense against pathogens. Therefore, understanding the mechanism underlying such a phenomenon in CLL seems to be of great importance. Here we discuss the recent reports analyzing the phenotype and functions of neutrophils in CLL, the most common leukemia in adults. We summarize the data concerning both the phenotype and the mechanisms by which neutrophils directly support the proliferation and survival of malignant B cells.

## 1. Introduction

Neutrophils for decades were considered to be ‘only’ simple effector cells and placed at the bottom of the immune response hierarchy. However, most recent evidence began to change this view by uncovering sophisticated mechanisms used by neutrophils as well as unique and unexpected functions of these cells [1]. Neutrophils, a type of myeloid leukocytes, are the most essential executives of the innate arm of the immune response. These cells are considered to be the most abundant type of white blood cells in mammals and first defenders against a large range of invading pathogens, including bacteria, fungi, protozoa, and viruses. Neutrophils are characterized as cells containing a large, segmented nucleus and various types of granules, which are specialized vesicles containing many toxic molecules enabling them to fulfil their antimicrobial function. These cells mature in the bone marrow and exist in the bloodstream for a short period of time. Neutrophils are recruited from the circulation to the site of infection by bacterial-derived stimulators, cytokines, and chemokine gradient. They eliminate microbes through various mechanisms including phagocytosis, production of reactive oxygen species, degranulation, and neutrophil extracellular traps (NETs) formation [2].

It has been shown that neutrophils, apart from antimicrobial properties, are capable of modulating the adaptive immune response at numerous levels, including B cells in the marginal zone, plasmacytoid dendritic cells, T cells, and NK cells. Moreover, neutrophils play a crucial role in a broad range of physiological and pathological processes, also beyond the immune system, such as chronic inflammation, autoimmune diseases, diabetes, thrombus formation, and cancers [3,4].

Over the past decade, the interest in the importance of the role of neutrophils in cancer has significantly increased. Importantly, the progression of cancerous diseases is associated with tumor cells and also with other key elements including recruited immune cells, their released pro-inflammatory factors, and the extracellular matrix, which constitute the tumor microenvironment. Most recent studies indicate that neutrophils represent a substantial part of the tumor microenvironment, which significantly affects cancer development, the therapeutic response, and the overall outcome of the disease. Neutrophils are recruited to the site of the tumor bed by the interplay of different chemokines that are responsible for their maturation, migration, proliferation, and function, including CXCR2 (interleukin 8 receptor, beta) granulocyte-colony stimulating factor (G-CSF) and interleukin 17A (IL-17A) [5,6,7].

Interestingly, for a long time neutrophils were considered as terminally differentiated cells. However, the studies on tumor-associated neutrophils (TANs) revealed that nothing could be more wrong. It occurred that neutrophils characterize high heterogeneity and phenotype plasticity [8]. Indeed, neutrophils respond to various mediators released in the tumor microenvironment resulting in their activation to either anti-tumor or pro-tumor phenotypes. Those two types of neutrophils were named N1 (anti-tumor neutrophil) and N2 (pro-tumor neutrophil), similarly to the classification of tumor-associated macrophages [9]. The neutrophil plasticity is evident when N1 or N2 can be shifted to each other, upon the activity of specific tumor-derived factors, such as transforming growth factor β (TGFβ), interferon β (IFN-β), IL-35, or a concentration of cytokines and oxygen in the tumor microenvironment [9,10,11,12].

N2 neutrophils play a pro-tumor role through multiple mechanisms. They promote tumor initiation, by the production of reactive oxygen species (ROS) [5] and reactive nitrogen species (RNS), as well as the proliferation of tumor cells by the secretion of neutrophil elastase (NE) and matrix metalloproteinase 9 (MMP9) [13]. Neutrophils can also support tumor angiogenesis by producing vascular endothelial growth factor (VEGF), prokineticin 2 (PROK2), and MMP9 [14,15], whereas cathepsin G, a neutrophil-derived serine protease, enables the formation of tumor cell aggregates that can further spread to distant sites leading to the development of metastases [10,16]. In addition, tumor progression can be achieved by attenuating the immune system, specifically T lymphocyte anti-tumor response, through a high expression of arginase 1 (ARG1) and inducible nitric oxide synthase (iNOS) [9,17]. In contrast, N1 neutrophils characterize high expressions of immuno-activating chemokines and cytokines including tumor necrosis factor α (TNF-α), intercellular adhesion molecule 1 (ICAM-1), and Fas. N1 anti-tumor activity is associated with direct tumor cell killing, by producing ROS and nitric oxide, or induction of Fas/TRAIL-related apoptosis. N1 neutrophils also generate hydrogen peroxide to inhibit the metastatic process. Other mechanisms include antibody-dependent cell-mediated cytotoxicity (ADCC) and activation of T cell function [7].

Most of the data describing TANs come from the analyses of solid tumors, whereas studies of these cells in hematological diseases are limited. Chronic lymphocytic leukemia (CLL), the most frequent leukemia in the USA and Europe, is characterized by the massive accumulation of mature CD5+ B cells in the bone marrow, blood, and lymph nodes [18]. The B-CLL gene expressing profile reflects memory B cells with active regulatory functions [19]. The high activation status of B cell receptor (BCR) signaling is crucial for clonal expansion and progression of CLL cells [20]. Thus, small-molecule compounds that inhibit BCR-associated kinases: Bruton’s tyrosine kinase, phosphoinositide 3-kinase (PI3K) delta, or spleen tyrosine kinase, were shown to be an effective treatment [21]. Nevertheless, CLL patients are suffering from frequent infections often leading to complications affecting chemo- or immunotherapy, which is a major cause of high morbidity and mortality rates in CLL patients [22]. Moreover, it was reported that between 2–9 percent of patients with CLL develop a Richter transformation (RT), a highly aggressive phenotype of the disease [23]. In RT the acquisition of multiple genetic defects including NOTCH1, trisomy 12, and an IGHV4-39 stereotypic BCR in B-CLL cells leads to a transformation that facilitates the rapid proliferation of leukemic cells [24]. Based on histopathological features such as the presence of enlarged B cells, the majority of RT cases occur as diffuse large B-cell lymphoma (DLBCL) [25]. Similar to CLL, DLBCL is also an aggressive neoplasm, derived from experienced B cells. Yet, DLBCL-type RT is characterized by a worse response to chemo- and immunotherapy when compared to de novo DLBCL [23]. Among several disturbances caused by intensive chemotherapy, neutropenia is one of the described problems in patients with DLBCL type of RT [26].

A high frequency of infections in CLL patients is associated with dysregulation of both adaptive and innate immune responses [27,28]. Among various dysfunctions of the immune system, a growing body of data presents the exhaustion of T cells, an increased number of T regulatory cells, hypogammaglobulinemia, and defects in the complement system that also results in the limited outcome of different immunotherapy [29,30,31]. The impaired immune response manifests also in the reduced efficacy of vaccinations, increased risk of skin cancers, and other neoplasms [32]. Recent reports suggest that CLL-related neutrophils are also responsible for this dysregulation of the immune system as they constitute the first line of defense against pathogens [33]. It is well documented that patients suffering from CLL characterize neutropenia and moreover, that neutrophils in patients with CLL had diminished response against bacteria [34]. In this study, we discuss the phenotype and functions of neutrophils in CLL as well as DLBCL, which phenotypically represents around 90 to 95 percent of RT cases [26].

## 2. Neutrophils Recruitment and Phenotype in CLL

Unlike the other cells in the immune system, neutrophils are released from the bone marrow as fully functional cells, able to recognize and neutralize the pathogens and support the development of an adaptive immune response [35]. Yet, in cancer, N2-type granulocytes enter the blood circulation before the termination of the maturation process and both function and phenotype of these cells can be further modified. Although the decreased number of neutrophils is accompanied by advanced disease or aggressive chemotherapy [36], the neutrophils still enriched the tumor microenvironment due to response to several cytokines, which role have been investigated in CLL and DLBCL (Table 1). For example, IL-8 and IL-17A are inflammatory factors that can be secreted by the tumor microenvironment or malignant cells and attract neutrophils to tumor niches [10,37,38]. Additionally, cognate receptors are also implicated in neutrophils activity and functions [39]. In the Eμ-TCL1 murine model of CLL, it was indicated that the stroma cells from the leukemia microenvironment overexpressed genes related to the chemotaxis of neutrophils, including chemotactic factors (S100A8, S100A9), receptors (CXCR2), inflammatory cytokines (IL-1beta), and integrins (Itgam/CD11b) [40]. In general, the CXCR2 influences the activation status of neutrophils and can be modulated by environmental factors, including TNFα or nitric oxide [41]. As the concentration of TNFα is increased in CLL patients’ serum and correlates with disease progression and survival it also may contribute to CXCR2 expression level [42].

The main membrane-bound markers that are associated with neutrophils functions include CD11b, CD64 (Fc γ receptor I), and CD54 (intracellular adhesion molecule-1, ICAM-1). The upregulation of CD64 and CD54 was observed in neutrophils isolated from patients with progressing CLL and it was related to low total number and percentage of circulating neutrophils [52]. The increase of CD64 is usually induced by neutrophil activation and it is associated with cytotoxic capacity and oxidative burst [53]. Upregulation of CD64 on neutrophils may occur also in response to IFNγ, which is elevated in the serum of CLL patients [54]. Therefore, the Manukyan and colleges suggested that the activated phenotype of neutrophils is a result of tumor-related cytokine secretion and may positively contribute to anti-CD20 therapy-induced phagocytosis [52]. Nevertheless, this phenotype of constantly activated neutrophils does not explain the reduced ability of neutrophils to neutralize pathogens in both CLL and DLBCL group of patients.

Indeed, CLL-derived neutrophils show to be resistant to LPS-induced upregulation of proinflammatory cytokines including IL-1β and tumor necrosis factor α (TNFα). Moreover, the stimulation with LPS did not affect the expression level of CD54, CD11b, CD62L, and CD64, but it lead to downregulation of toll-like receptor (TLR) 2 [52].

Interestingly, the leukemia-derived neutrophils show to be more resistant to apoptosis in ex vivo conditions when cultured in the presence of G-CSF. Similarly, the neutrophils isolated from CLL human patients display a higher survival [33]. The pro-survival influence of G-CSF and GM-CSF on neutrophils was described in different tumors, including gastric cancer [55,56] and pulmonary adenocarcinoma [57]. Moreover, in breast cancer, the tumor cells-derived GM-CSF was shown to induce the ARG1 expression in the myeloid cells through STAT3 (signal transducer and activator of transcription) and p38 MAPK (mitogen-activated protein kinases) signaling pathway [58]. In line with these reports, the depletion of G-CSF and GM-CSF in CLL-conditional medium reduces the survival capacity of neutrophils [33]. Both G-CSF and GM-CSF are produced by CLL cells [59]. On the other hand it is worth mentioning that the effect of GM-CSF is pleiotropic and this cytokine is also used as a therapy. GM-CSF can improve the efficacy of immunotherapy of CLL [60] as well as enhances CHOP (cyclophosphamide/doxorubicin/vincristine/prednisone) and R-CHOP (Rituximab + CHOP) treatment in mouse model of DLBCL [61]. However, the influence of such therapies on neutrophils function has not been evaluated yet.

## 3. Interactions of Neutrophils with Malignant B Cells

The B cell-derived immune response can be regulated by the neutrophils. These cells are responsible for the transportation of bacterial antigens to splenic marginal zones that support B cells in the development of T cell-independent immune response [62]. Moreover, due to the secretion of B-cell activating factor (BAFF), neutrophils promote B cell maturation and survival [63]. BAFF activates in B cells several downstream pathways, including non-canonical NF-κB-dependent pathway, PI3K, and the protein kinase B/mammalian target of rapamycin (AKT/mTOR) signaling leading to metabolic reprogramming of B cells, increasing the cell lifespan [64]. It was also reported that reprogramming of neutrophils executed by IL-10 transforms neutrophils into B-helper cells that induce immunoglobulin class switching, hypermutation, and production of antibodies by B cells. IL-10-reprogramed neutrophils secrete also BAFF, APRIL, and IL-23. Interestingly APRIL in return leads to the secretion of IL-10 by B cells [65].

The secretion of APRIL by TANs was described in DLBCL [66]. TANs isolated from patients’ blood cooperated with stroma cells to promote the proliferation of leukemic cells via BAFF and APRIL-dependent mechanisms. In return, the stoma cells of DLBCL patients support the survival of neutrophils and can increase NETs formation of PMA (Phorbol myristate acetate)-induced neutrophils [46]. Moreover, the infiltration of neutrophils analyzed by the detection of ELANE, the gene encoding the neutrophil elastase, was associated with reduced overall survival of DLBCL patients [67].

In the Eμ-TCL1 mice, the presence of neutrophils in the leukemia microenvironment has been observed mainly in the red pulp as well as in the marginal zone of spleens, where they are infiltrating the malignant tissue. It was reported that tumor-infiltrating neutrophils secrete survival cytokines including APRIL and BAFF, which are important for B cells proliferation [40]. However, the mRNA encoding those two proteins was not elevated in neutrophils isolated from CLL patients when compared to healthy donors. This effect may be a result of analyzing the cells isolated from peripheral blood which does not represent the leukemia microenvironment.

Depletion of neutrophils in mouse Eμ-TCL1 model, with already developed CLL, twice reduced the leukemia burden in mouse spleens [40]. Additionally, the depletion of the Ly6G+ population affected leukemic cells by reduction of S-phase (Brdu+) of cell cycle and therefore limits their potential to proliferate. Interestingly, lack of neutrophils leads also to overexpression of early growth response 1 (Egr1) and 2 (Egr2) transcription factors, important for B cells development, causing the cell-cycle arrest at the G2–M phase [40]. Obtained results clearly show that neutrophils support leukemia progression in the Eμ-TCL1 mice.

Finally, Podaza et al. presented data showing that NETs produced by CLL neutrophils enhance the expression of CD80, CD86 and CD69 on leukemic cells and delay their spontaneous apoptosis in in vitro conditions [68]. In SPARC (secreted protein acidic rich in cysteine) knockout mice (Sparc−/−), the phenotype of which is prototypical for CLL and associated with overt lymphomagenesis, the increased NETs formation, BAFF, and IL-21 secretion and elevated resistance to apoptosis were observed [69]. Moreover, it was reported that NETs in Sparc−/− mice supports the in vitro proliferation of CD5+ B cells via NF-κB activation and was abolished by the addition of DNAse. Remodeling of the extracellular microenvironment in Sparc−/− affects neutrophils that provide the auto-antigen stimulation for B-cells and favor the transformation of CD5+ B cells [69].

## 4. The Mechanisms of Immunosuppression Derived by Neutrophils in CLL

In general, neutrophils can affect the adaptive immune response via direct interactions, or by the release of a variety of mediators [70]. The formation of an “immune synapse” between neutrophils and T cells may result in T cells suppression and apoptosis or, on the contrary, may activate T cells by antigen presentation [71,72]. It was also suggested that the release of NETs may lead to apoptosis of both CD8+ and CD4+ T cells [73]. The synthesis of well described immunosuppressive factors, like 2-3-dioxygenase indoleamine, ARG1 or myeloperoxidase affects the adaptive immune response [74,75,76,77]. Finally, in solid tumors the CD16highCD62Ldim subpopulation of neutrophils was shown to exert immunosuppressive activity on T cells [78,79]. Due to the fact that CD62Ldim presents a unique profile of protein secretion, it has been proposed that CD62Ldim neutrophils are a separate subset of cells [80] that respond differently to lipopolysaccharide (LPS) when compared to the CD62Lbright subpopulation [79].

The CD16highCD62Ldim subset of neutrophils had been described in the blood of CLL patients and the formation of this subpopulation was shown to be a result of the secretion of IL-10 by the leukemic cells. Additionally, the leukemic cells-derived G-CSF and GM-CSF increased the lifespan of neutrophils through the upregulation of antiapoptotic factor Bfl-1 [33]. Moreover, the neutrophils isolated from CLL patients are able to more effectively inhibit PHA-induced activation of T cells than neutrophils from healthy donors [33]. Nevertheless, the direct impact of neutrophils on adaptive immune response in CLL and DLBCL needs further evaluation.

## 5. Defects in Neutrophils’ Functions and Their Influence on Immune Response Development in CLL and DLBCL

Neutrophils isolated from CLL patients revealed dysfunction in the phagocytic killing of key non-opsonized bacterial (Staphylococcus aureus and Pseudomonas aeruginosa), while no difference in activity against fungal pathogens (Candida albicans and Aspergillus fumigatus) was observed [34]. Interestingly, the neutrophils phenotype and phagocytosis activity can be changed by immunotherapy. It was shown that glycoengineered antibodies against CD20 (Obinutuzumab) induce CLL cells phagocytosis by neutrophils, which was accompanied by the increase of CD11b and reduction of CD62L expression [81].

The ability to ROS generation by CLL neutrophils was also measured. Stimulation with fMLP (N-Formylmethionine-leucyl-phenylalanine) or PMA intensifies ROS generation in CLL neutrophils compared to healthy controls [52]. However, when the concentration of ROS was analyzed in neutrophils isolated from bone marrow of Eμ-TCL1 mice, the ability to generate the H_2_O_2_ was similar in Eμ-TCL1 and control animals [40]. These results may underline the difference between human disease and mouse models or may suggest that some of the dysfunctions of neutrophils are mediated by the leukemia microenvironment.

Neutrophils isolated from CLL patients revealed reduced random migration when compared to healthy controls. Similarly, the fMLP and C5a-stimulated chemotaxis of neutrophils is also decreased in CLL. Moreover, the treatment against CHL (chlorambucil) did not restore the neutrophils ability to migrate [36].

The release of NETs can occur after stimulation with a variety of factors including microorganisms, TNFα, LPS, high mobility group B1, or in in vitro conditions with PMA. It was reported that neutrophils obtained from CLL patients, stimulated with PMA or a mix of TNFα and LPS, release a significantly higher level of NETs compared to those from healthy, age-matched donors [68]. Moreover, the incubation of PMA-prestimulated neutrophils with plasma isolated form CLL patients again enhanced expression of CXCR2 and production of NETs leading to an increase of NETs-related death of cells. Additionally, IL-8 was pointed out as a key factor that prime CLL neutrophils to release NETs [68].

In DLBCL the level of NETs was significantly elevated and is associated with advanced stage of disease as well as poor prognosis [82]. Similar to CLL, NETs are induced by IL-8 and the inhibition of IL-8 receptor reduced tumor growth in the DLBCL mouse model. However, in DLBCL NETs promote the survival of malignant B cells via TLR9-dependent manner [82]. In line with this observation the TLR9 antagonist was shown to be effective in reducing the growth of B-cell lymphoma [83].

## 6. Conclusions

**Figure 1:**
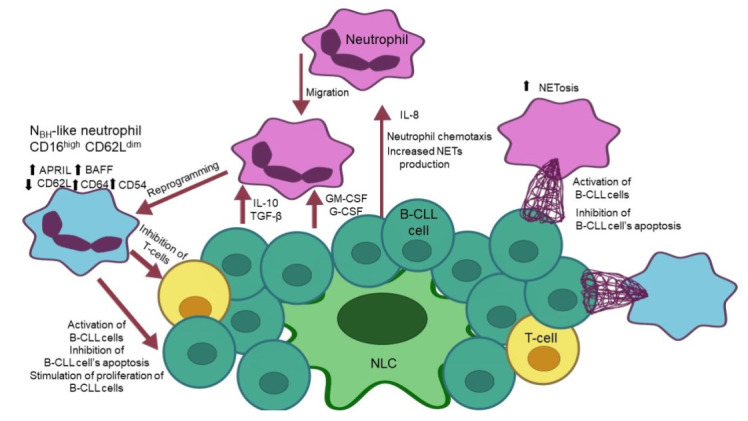
The summary of molecular and phenotypic changes that neutrophils undergo in proliferation centers of CLL. Secretion of IL-8 in the proliferation center (PC) causes neutrophil chemotaxis, resulting in their migration to PC and increased NETs production. Malignant B lymphocytes and nurse-like cells (NLC) produce G-CSF, GM-CSF, IL-10, and TGF-β leading to reprogramming of neutrophils to B-CLL helper-like neutrophils (NBH-like). Reprogrammed neutrophils became B cell helper like neutrophils CD16 high CD62L dim. Expression of APRIL, BAFF, CD64, and CD54 is upregulated, while expression of CD62L is downregulated. Neutrophils cause the activation of B-CLL cells, inhibition of their apoptosis and stimulation of cells’ proliferation. NETs formation in CLL PC is increased, which results in activation of B-CLL cells and inhibition of their apoptosis.

The dual nature of the neutrophils may favor the tumor progression or, to the contrary, could be important for the effectiveness of the antitumor immune response and used therapy. In the case of lymphoma and leukemia neutrophils were shown to contribute to the efficacy of the treatment with rituximab and obinutuzumab [81,84]. However, in CLL and DLBCL neutrophils with a unique phenotype are attracted to the tumor microenvironment and produce factors important for malignant B cell proliferation and survival. The phenotype of these cells is affected by the leukemia microenvironment and polarizes neutrophils into leukemia helping cells (Figure 1). Thus, it is not surprising that a number of studies indicate that neutrophils are important in the CLL progression. Moreover, it is suggested that the interaction of neutrophils with the extracellular matrix may also contribute to leukemia development. Simultaneously, the defects of neutrophils, which prevent their proper response to pathogens, are emphasized. The reduced ability to migrate, phagocytosis, and defective response to LPS are the basis of the impaired defense against pathogens. Altogether these facts present the neutrophils as a crucial element of the CLL microenvironment that influences both disease progression and patient survival. Nevertheless, still little is known about neutrophils’ influence on development of anti-leukemia immune response.

## Figures and Tables

**Table 1:** Cytokines and receptors secreted in the microenvironment of leukemia and/or lymphoma that may be responsible for neutrophils’ recruitment.

Cytokine	Role in Neutrophils Recruitment	Refs.
IL-8	In CLL IL-8 is secreted by leukemia-associated monocytes and macrophages. In DLBCL both malignant and stroma cells seem to be the source of this chemokine. The elevated secretion of IL-8 was described as a prognostic marker that correlates with Rai stage and beta-2 microglobulin factors and is associated with the high risk of patient death in CLL. Additionally, in DLBCL upregulation of IL-8 correlates with the level of proliferation inducing ligand (APRIL) in patients’ serum.	[43,44,45,46,47,48]
IL-17A	Elevated in CLL patients’ serum. Unfavorable prognosis marker that promotes the resistance to rituximab in DLBCL. However, IL-17A has not been yet linked directly with the neutrophils infiltration in CLL and DLBCL.	[49,50,51]
CXCR2	Expression increased in bone marrow-derived neutrophils of Eμ-TCL1 murine model of CLL.	[40]
